# The Potent Complement Stimulus of Sanguineous Versus Crystalloid Cardiopulmonary Bypass Prime during Pediatric Cardiac Surgery

**DOI:** 10.1177/21501351261418297

**Published:** 2026-03-14

**Authors:** Joel David Bierer, Roger Stanzel, Mark Henderson, John Sapp, Pantelis Andreou, Jean S. Marshall, David Horne

**Affiliations:** 1 Division of Cardiac Surgery, 3688 Dalhousie University, Halifax, Nova Scotia, Canada; 2 Department of Clinical Perfusion, 432234 Nova Scotia Health Authority, Halifax, Nova Scotia, Canada; 3 Division of Cardiology, 3688 Dalhousie University, Halifax, Nova Scotia, Canada; 4 Department of Community Health & Epidemiology, 3688 Dalhousie University, Halifax, Nova Scotia, Canada; 5 Department of Microbiology & Immunology, 3688 Dalhousie University, Halifax, Nova Scotia, Canada

**Keywords:** pediatric cardiac surgery, congenital heart disease, cardiopulmonary bypass, complement, cytokine, chemokine, inflammation

## Abstract

**Background:** Sanguineous preparation of the cardiopulmonary bypass (CPB) circuit with allogeneic blood products is known to contain substantial complement mediator burden. This study aims to assess the longitudinal immunologic impacts during pediatric CPB. **Methods:** In this post-hoc analysis of a prospective observational cohort study, 40 pediatric patients undergoing cardiac surgery with CPB were grouped by CPB prime type indicated by standard of care (*sanguineous* vs *crystalloid*). Arterial samples were collected before CPB, after CPB initiation, and at 30-min intervals until weaning or 180 min. Luminex^®^ measured concentrations of 33 inflammatory mediators for time series and fold change comparison between groups. **Results:** The *sanguineous prime* group (n = 26) was younger (4.0 [0.2-6.0] versus 48.5 [39.0-69.5] months; *P* < .001) and smaller (4.9 [3.4-6.6] versus 17.2 [14.9-19.6] kg; *P* < .001) than the *crystalloid prime* group (n = 14). The *sanguineous* group had significantly more circulating complement mediators, including C3a, C3b, and C5a, and soluble adhesion molecules throughout the CPB time series (*P* < .05). TNF, IL-1α, and IL-1β were relatively static in both groups, although slightly more prominent in the *crystalloid* group (*P* < .05). IL-6, IL-10, and CXCL8 profiles were comparable between groups. **Conclusions:** Patients who receive sanguineous CPB prime have elevated complement and soluble cellular adhesion molecule burden throughout CPB, relative to a crystalloid prime. Therefore, these allogeneic preparations should be considered an immunogenic stimulus during pediatric CPB, and future innovation should focus on less inciting alternatives.

## Introduction

Sanguineous prime of the cardiopulmonary bypass (CPB) circuit, consisting of allogeneic fresh frozen plasma (FFP) and red blood cells (RBC), has long been considered the standard of care for neonates and small infants undergoing open heart surgery.^1–3^ The primary purpose of this prime preparation is to mitigate the adverse effects of hemodilution, which arises from a significant disparity between the body's circulating volume and the CPB circuit volume in small patients, on oxygen-carrying hemoglobin and coagulation factors relevant to perioperative bleeding and transfusion.^1–4^ Unfortunately, sanguineous primes are associated with several adverse effects, including supraphysiologic inflammatory complement concentrations from the FFP along with RBC storage lesions.^5,6^ Although causality has not been proven, there could potentially be an unexplored relationship between sanguineous prime and adverse clinical outcomes, as neonates and infants carry the highest rate of morbidity and mortality following cardiac surgery.^7^ Even allowing for the greater severity of congenital heart disease and corresponding risk of operative repair in this group, this observation of a potentially modifiable risk factor warrants further investigation to improve outcomes in this vulnerable patient population.

Although the inflammatory reaction during cardiac surgery and CPB is multifactorial and complex, complement system activation and anaphylatoxins appear to be associated with postoperative morbidities following children's heart surgery.^8–12^ The sanguineous prime preparation process yields a mixture of elevated complement concentrations—particularly the activated components C3a, C3b, and C5a—which is directly transferred to the patients upon CPB initiation.^6,13^ Conversely, cytokine and chemokine concentrations in the prime appear to be less than patient baseline values before CPB, perhaps influenced by buffered ultrafiltration (BUF) of the prime.^13–18^ Sanguineous prime could be a key modifiable risk factor for activation of the complement reaction and inflammatory cascade relevant to postoperative morbidities. The objective of this retrospective cohort study was to contrast the immunologic profile of patients receiving sanguineous prime to those receiving crystalloid prime throughout the duration of CPB.

## Methods

### Study Design

This is a post-hoc cohort analysis using data from a single-center single-arm prospective clinical trial (NCT05154864 on ClinicalTrials.gov) investigating the relationship between circulating inflammatory mediators and postoperative clinical outcomes in pediatric patients undergoing cardiac surgery with CPB and continuous ultrafiltration (n = 40). The primary clinical results of this patient cohort are published separately.^11^ In this cohort sub-analysis, the exposure of interest for comparative analysis is CPB prime composition: *sanguineous prime* (n = 26) versus *crystalloid prime* (n = 14). The outcomes of interest are inflammatory mediators (complement, cytokines, chemokines, and adhesion molecules) in a time series from before CPB initiation, until the first of CPB cessation or 180 min of CPB. Inflammatory mediator data after 180 min were excluded from this analysis due to insufficient sample size. The study is reported following the Strengthening the Reporting of Observational Studies in Epidemiology (STROBE) guidelines (Supplemental Table 1).^19^

### Data Sources

Written informed consent was obtained from substitute decision-makers for all participants under a protocol approved by the IWK Health Centre Research Ethics Board (#1024869) on November 21, 2019. Patients were prospectively enrolled and completed the study protocol between August 2020 and June 2021 at the IWK Health Centre in Halifax, Nova Scotia, Canada. Patient baseline demographics and intraoperative data were collected. Samples (1 ml) of the sanguineous prime were taken after BUF and prime preparation completion. Arterial blood (1 mL) was drawn post-sternotomy but before CPB initiation (Pre-CPB baseline), once full-flow CPB was reached (CPB initiation), then every 30 min until 180 min of CPB were reached. There was no imputation of missing data, loss-to-follow-up, or sensitivity analyses.

### Cardiopulmonary Bypass Prime Preparation

Cardiopulmonary bypass prime composition was according to the institutional standard of care. Sanguineous prime—consisting of unwashed 200 ml packed RBC, 200 ml FFP, 300 ml of Plasma-Lyte^®^ A and 300 ml of 0.45% saline—was used for patients < 10 kg. The mixture underwent BUF to normalize metabolic parameters and hemoconcentrate the volume to approximately 500 ml.^14^ A detailed descriptive analysis on the sanguineous prime preparation process with biochemical and immunologic data has been published separately.^6^ Crystalloid prime was used for those > 10 kg and included 1000 ml Plasma-Lyte^®^ A and 500 ml 5% albumin, which was reduced to roughly 700 ml. Retrograde autologous priming was considered for patients in the crystalloid group. Heparin, sodium bicarbonate, calcium chloride, magnesium sulphate, furosemide, and mannitol were applied at standard weight-based doses. A *Liva Nova S5^TM^* CPB System with phosphorylcholine coating (48-40-00, London, UK), *Terumo FX05* (weight < 10 kg) or *FX15* (weight > 10 kg) oxygenators (1CX*FX05RE/1CX*FX15E, Tokyo, Japan) and *Terumo Capiox^®^* Hemoconcentrator HCO5 (1CX*HC05S, Tokyo, Japan) were used. All patients received standard of care CPB conduct, including continuous subzero balance ultrafiltration with effluent extraction at 30 ml/kg/h and physiologic volume replacement at 25 ml/kg/h.^20^ Hematologic resuscitation in both groups with pRBC during CPB was at the discretion of the intraoperative team for optimal oxygen delivery on CPB.

### Immunoanalysis

*Luminex* immunoanalysis of patient samples was completed with a *Bio-Rad Bio-Plex^®^ 200* System (Hercules, CA, USA). Thirty-three prespecified human inflammatory factors were analyzed in biologic samples via: *ThermoFisher* C3a Simplex Kit (EPX010-12282-901, Waltham, MA, USA), *Millipore Sigma* Human Complement Magnetic Bead Panel 1 (HCMP1MAG-19K-05, Burlington, MA, USA), *Millipore Sigma* Human Complement Magnetic Bead Panel 2 (HCMP2MAG-19K-06, Burlington, MA, USA), *BioTechne R&D Systems* Human XL Cytokine Luminex Performance Panel (FCSTM18-21, Minneapolis, MN, USA), *BioTechne R&D Systems* Human Magnetic Luminex Assay (LXSAHM-05, Minneapolis, MN, USA), and *BioTechne R&D Systems* Human Magnetic Luminex Assay (LXSAHM-01, Minneapolis, MN, USA). Bio-Rad Bio-Plex^®^ Manager^TM^ Software 6.2 (Hercules, CA, USA) was used to complete the data acquisition and used Logistic-5PL regression for all analytes. All assays were conducted according to the manufacturer's instructions. Mediator concentrations were standardized via the global median ratio method—where sample *x* from mediator assay *a* was corrected by ([mediator]*_xa_* * ([mediator]_global median_ / [mediator]_assay *a* median)_)—to account for inter-assay variability.

### Statistical Analysis

Categorical variables are reported as numbers (%), and continuous variables are presented as medians and interquartile range (IQR). Measured mediator concentrations (*x*g/ml) were converted to mediator mass (*x*g/kg) to account for vital physiological differences in circulating blood volume and relative CPB circuit size, which differ between neonates, infants, and children. CPB circuit volume was measured, and the patient's circulating volume was 90 ml/kg for neonates, 80 ml/kg for infants, and 70 ml/kg for children.^21^

Time series for each mediator were generated to describe the longitudinal inflammatory burden and presented as median (IQR). The area under the curve (AUC) was calculated for each mediator to estimate the entire exposure burden through the time series, separately in the *sanguineous* and *crystalloid prime* groups, using trapezoidal integration of the median time series curve through 180 min.^22,23^ Individual AUC for each patient was not used because of shorter CPB times in the *crystalloid prime* group associated with biased results. Fold changes (FC) between Pre-CPB baseline and CPB cessation or 180 min of CPB (End-CPB) were calculated by *([mediator]_End-CPB_ – [mediator]_Pre-CPB_) / [mediator]_Pre-CPB_* and presented as median fold change with [95% CI] estimated by 1000 nonparametric bootstrap samples with adjusted percentile interval.^24^ Comparisons between *sanguineous* and *crystalloid prime* groups were made using the Wilcoxon rank sum test. Statistical analyses were performed using R (version 4.4.2, *R Foundation for Statistical Computing*, Vienna, Austria) with α = 0.05.

## Results

### Participants

A total of 49 consecutive patients were screened during the study period, and 40 patients (80%) gave informed consent to participate. Nine of 49 patients (18%) were not enrolled due to a genetic syndrome with severe multi-organ abnormalities (n = 3, 6%), weight over 30 kg (n = 3, 6%), logistical process restraints (n = 2, 4%), and refusal to participate (n = 1, 2%). All 40 enrolled patients completed data collection and are included in this post-hoc analysis with no exclusions. The patients were divided into 2 groups based on CPB prime indicated by standard of care; 26 patients received sanguineous CPB prime, and 14 patients received crystalloid CPB prime.

Baseline characteristics of the group are summarized in Table 1. The *sanguineous prime* patients were significantly younger, had a lower weight, a higher proportion of STAT 3 and 4 procedures, but no difference in sex distribution, compared with the *crystalloid prime* group. The pre-CPB baseline concentrations of C1q, C2, C3, C3a, C3b, C4, C4b, C5, C5a, CFB, CFH, IL-1α, IL-1β, IL-2, IL-10, ET1, GM-CSF, CCL2, CCL4, CXCL1, CXCL2, L-selectin, P-selectin, and ICAM-1were comparable between groups (*P* > .05). CFI mediator mass (*P* = 1.4 × 10^−4^) was statistically higher in the crystalloid group while the *sanguineous prime* group showed higher levels of TNF (*P* = .005), IL-1Ra (*P* = .005), IL-6 (*P* = .003), TRAIL (*P* = .007), CCL3 (*P* = .01), CXCL8 (*P* = .01), E-selectin (*P* = .02), and VCAM-1 (*P* = .02).

**Table 1.** Patient Demographics.

	Sanguineous prime (n = 26)	Crystalloid prime (n = 14)	*P*-value
Age (months)	4.0 (0.2-6.0)	48.5 (39.0-69.5)	<.001
Neonate	10 (39%)	0 (0%)	<.001
Infant	12 (46%)	0 (0%)	
Child	4 (15%)	14 (100%)	
Male sex	17 (65%)	6 (43%)	.09
Weight (kg)	4.9 (3.4-6.6)	17.2 (14.9-19.6)	<.001
Body surface area (m^2^)	0.29 (0.22-0.35)	0.74 (0.4-0.85)	<.001
STAT Score			.002
STAT 1	6 (23%)	7 (50%)	
STAT 2	5 (19%)	7 (50%)	
STAT 3	2 (8%)	0 (0%)	
STAT 4	13 (50%)	0 (0%)	
Baseline inflammatory mediator mass			
**Complement**			
C1q (mg/kg)	3.9 (2.9-5.7)	4.2 (2.9-5.8)	.94
C2 (mg/kg)	0.04 (0.02-0.06)	0.02 (0.02-0.04)	.07
C3 (mg/kg)	1.2 (0.9-1.6)	1.1 (0.7-1.4)	.29
C3a (ug/kg)	1.4 (0.8-1.9)	1.2 (0.8-1.5)	.27
C3b (mg/kg)	0.6 (0.1-3.3)	0.3 (0.1-1.6)	.26
C4 (mg/kg)	12.7 (10.4-15.3)	12.3 (10.5-16.1)	.79
C4b (mg/kg)	0.9 (0.8-1.0)	1.0 (0.9-1.1)	.12
C5 (mg/kg)	1.0 (0.8-1.4)	1.0 (0.7-1.17)	.92
C5a (ng/kg)	1.8 (0.9-4.6)	1.6 (1.0-6.8)	.70
CFB (mg/kg)	11.7 (8.7-13.4)	11.0 (9.0-15.2)	.83
CFH (mg/kg)	14.4 (11.3-18.8)	15.9 (11.4-19.4)	.77
CFI (mg/kg)	1.5 (1.2-1.8)	2.7 (2.1-3.2)	<.001
**Cytokines**			
TNF (ng/kg)	3.2 (2.1-3.8)	1.9 (1.5-2.3)	.005
IL-1α (ng/kg)	4.3 (3.5-5.8)	3.8 (2.6-4.6)	.16
IL-1β (ng/kg)	0.7 (0.4-1.2)	0.6 (0.3-0.9)	.25
IL-1Ra (ng/kg)	46.5 (34.8-89.3)	23.7 (20.4-30.1)	.003
IL-2 (ng/kg)	0.9 (0.7-1.3)	0.8 (0.5-0.9)	.11
IL-6 (ng/kg)	1.3 (0.9-2.4)	0.5 (0.1-0.8)	.003
IL-10 (ng/kg)	5.3 (3.5-8.2)	4.6 (2.6-6.6)	.54
ET1 (ng/kg)	0.13 (0.02-0.23)	0.11 (0.03-0.11)	.16
GM-CSF (ng/kg)	1.3 (1.0-2.2)	1.4 (0.9-2.3)	.63
TRAIL (ng/kg)	14.3 (12.4-18.1)	10.4 (8.1-13.5)	.007
**Chemokines**			
CCL2 (ug/kg)	20.1 (11.9-22.8)	14.3 (10.0-17.4)	.13
CCL3 (ng/kg)	2.9 (2.3-3.4)	2.3 (1.8-2.6)	.01
CCL4 (ug/kg)	44.7 (33.0-53.5)	36.7 (26.5-44.9)	.12
CXCL1 (ng/kg)	14.3 (11.1-18.1)	10.1 (7.0-15.9)	.06
CXCL2 (ug/kg)	0.14 (0.10-0.23)	0.14 (0.08-0.19)	.66
CXCL8 (ng/kg)	1.2 (0.8-1.7)	0.6 (0.5-1.0)	.01
**Adhesion Molecules**			
E-selectin (ug/kg)	4.6 (3.3-5.8)	2.7 (2.3-4.2)	.02
L-selectin (ug/kg)	43.5 (34.4-52.5)	46.4 (37.4-55.6)	.62
P-selectin (ug/kg)	2.8 (2.2-3.3)	2.6 (2.3-3.0)	.56
ICAM-1 (ug/kg)	21.7 (18.4-25.7)	19.2 (16.7-22.4)	.20
VCAM-1 (ug/kg)	7.8 (5.7-12.2)	5.4 (4.9-6.3)	.01

Abbreviations: C, complement; CF, complement factor; CCL, C-C motif chemokine ligand; CXCL, C-X-C motif chemokine ligand; CPB, cardiopulmonary bypass; IL, interleukin; STAT, Society of Thoracic Surgeons-European Association for Cardio-Thoracic Surgery; TNF, tumor necrosis factor.

### CPB Prime Composition

The biochemical and immunologic characteristics between sanguineous and crystalloid prime are inherently different (Table 2). The sanguineous prime contained C1q, C2, C3, C3a, C3b, C4, C4b, C5, C5a, CFB, CFH, and CFI mediator mass that was in excess of the patient's pre-CPB baseline (*P* < .05). Conversely, the sanguineous prime contained TNF, IL-1a, IL-1B, IL-1Ra, IL-2, IL-6, IL-10, CCL3, CCL4, CXCL1, CXCL2, CXCL8, GM-CSF, TRAIL, and VCAM-1 mediator mass that was less than that of the patient's pre-CPB baseline (*P* < .05). CCL2, ET1, E-selectin, L-selectin, P-selectin, and ICAM-1 were not different between the sanguineous prime and patient pre-CPB baseline (*P* > .05). The crystalloid prime did not contain inflammatory mediators as it did not include any allogeneic or recombinant blood products.

**Table 2.** CPB Prime Characteristics.

	Sanguineous prime (n = 26)	Crystalloid prime (n = 14)	*P*-value
CPB prime volume (ml)	500 (450-638)	742 (575-852)	<.001
Prime volume (ml/kg)	102 (80-150)	39 (34-46)	<.001
Hemoglobin (g/L)	107 (92-126)	NR	NA
pH	7.38 (7.33-7.41)	NR	NA
CO_2_ (mm Hg)	48 (46-49)	NR	NA
HCO_3_ (mM)	26 (23-30)	NR	NA
Lactate (mM)	2.2 (1.6-3.0)	NR	NA
CPB prime inflammatory mediator mass			
**Complement**			
C1q (mg/kg)	7.4 (5.1-12.3)	0	NA
C2 (mg/kg)	0.25 (0.17-0.36)	0	NA
C3 (mg/kg)	6.0 (4.1-10.3)	0	NA
C3a (ug/kg)	12.9 (8.6-18.8)	0	NA
C3b (mg/kg)	20.5 (9.4-69.2)	0	NA
C4 (mg/kg)	22.8 (15.8-34.4)	0	NA
C4b (mg/kg)	2.0 (1.5-2.6)	0	NA
C5 (mg/kg)	1.9 (1.2-2.9)	0	NA
C5a (ng/kg)	80.1 (34.6-226.4)	0	NA
CFB (mg/kg)	21.3 (12.3-26.4)	0	NA
CFH (mg/kg)	27.8 (16.6-42.9)	0	NA
CFI (mg/kg)	3.4 (2.6-6.5)	0	NA
**Cytokines**			
TNF (ng/kg)	0.17 (0.05-0.45)	0	NA
IL-1α (ng/kg)	0.42 (0.29-0.71)	0	NA
IL-1β (ng/kg)	0.05 (0.03-0.14)	0	NA
IL-1Ra (ng/kg)	26.7 (15.0-39.1)	0	NA
IL-2 (ng/kg)	0.24 (0.16-0.28)	0	NA
IL-6 (ng/kg)	0.12 (0.07-0.16)	0	NA
IL-10 (ng/kg)	0.5 (0.1-1.5)	0	NA
ET1 (ng/kg)	0.06 (0.02-0.25)	0	NA
GM-CSF (ng/kg)	0.33 (0.17-0.70)	0	NA
TRAIL (ng/kg)	4.7 (3.4-8.5)	0	NA
**Chemokines**			
CCL2 (ug/kg)	19.4 (13.5-35.9)	0	NA
CCL3 (ng/kg)	1.1 (0.4-1.5)	0	NA
CCL4 (ug/kg)	14.7 (9.0-20.8)	0	NA
CXCL1 (ng/kg)	3.3 (1.5-5.8)	0	NA
CXCL2 (ug/kg)	0.008 (0.004-0.013)	0	NA
CXCL8 (ng/kg)	0.17 (0.13-0.30)	0	NA
**Adhesion molecules**			
E-selectin (ug/kg)	3.2 (2.2-6.7)	0	NA
L-selectin (ug/kg)	47.4 (29.3-71.2)	0	NA
P-selectin (ug/kg)	3.1 (2.1-4.2)	0	NA
ICAM-1 (ug/kg)	23.1 (16.5-35.8)	0	NA
VCAM-1 (ug/kg)	5.5 (3.1-6.8)	0	NA

Abbreviations: BUF, buffer ultrafiltration of the prime; C, complement; CF, complement factor; CCL, C-C motif chemokine ligand; CXCL, C-X-C motif chemokine ligand; CPB, cardiopulmonary bypass; IL, interleukin; NA, not applicable; NR, not recorded; TNF, tumor necrosis factor.

### Operative Data

All patients received the indicated cardiac operation. Through 180 min of CPB, 13 of 26 *sanguineous prime* group patients remained perfused by CPB, while only 4 of 14 *crystalloid prime* group patients remained on CPB. The aortic cross clamp was removed at 132 (116-142) minutes of CPB in the *sanguineous* group and 92 (67-133) minutes of CPB in the *crystalloid* group. The total CPB time was 193 (141-278) minutes for the *sanguineous group* and 136 (92-181) minutes for the *crystalloid group,* while the total myocardial ischemia time was 94 (66-117) minutes for the *sanguineous group* and 68 (36-120) minutes for the *crystalloid group*. Seven of 26 patients (27%) in the *sanguineous group* and 1 patient of 14 (7%) in the *crystalloid group* underwent deep hypothermic circulatory arrest during their operation (*P* = .22). The lowest recorded temperature during CPB was 28 (22-32)°C and 32 (32-34)°C in the *sanguineous* and *crystalloid group* respectively (*P* = .001).

### Inflammation During Cardiopulmonary Bypass

The patient estimated circulating volumes during CPB were different between *sanguineous prime* and *crystalloid prime* groups with 150 (135-192) ml/kg and 102 (98-112) ml/kg (*P* < .001), respectively. This reflects the differential relationship between patient and CPB prime volumes across neonatal, infant, and child age groups.

The time series of complement mediator masses between *sanguineous* and *crystalloid prime* groups, along with time point comparisons and median AUC, are found in Figure 1. All complement mediators—C1q, C3, C3a, C3b, C4, C4b, C5, C5a, CFB, CFH, and CFI mediator masses were more prominent in the *sanguineous* group. The profiles of cytokine and chemokine mediators exhibited distinct characteristics. CCL2, CCL3, and TRAIL concentrations were more consistently higher in the sanguineous prime group, while CXCL2 and GM-CSF were generally higher in the *crystalloid prime* group. TNF, IL-1α, IL-1β, IL-1Ra, IL-2, IL-6, IL-10, ET1, CCL4, CXCL1, CXCL8 were similar between groups (Figures 2 and 3). The adhesion molecule masses of E-selectin, L-selectin, P-selectin, ICAM-1, and VCAM-1 were higher in the *sanguineous prime.*

**Figure 1.**
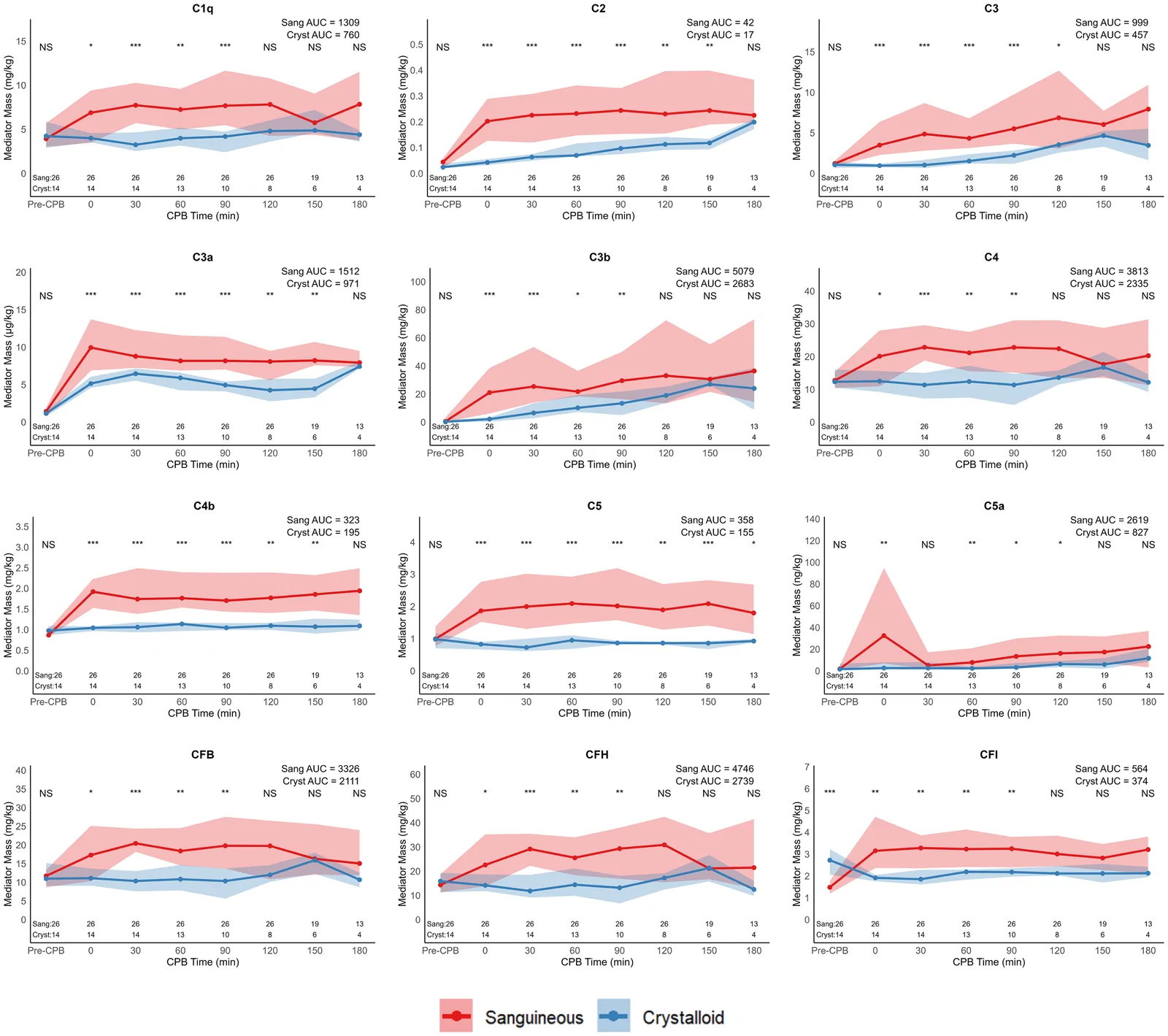
Complement mediator masses are elevated during CPB following sanguinous prime exposure. Values are displayed as median and interquartile range ribbon. Statistical comparisons between groups are by the Wilcoxon rank sum test at each time point: NS indicates *P* > .05, * indicates *P* < .05, ** indicates *P* < .01, and *** indicates *P* < .001. Observations (N) through the time series are recorded. Abbreviations: AUC, area under curve; C, complement; CF, complement factor; Cryst, crystalloid prime group; Sang, sanguineous prime group.

**Figure 2.**
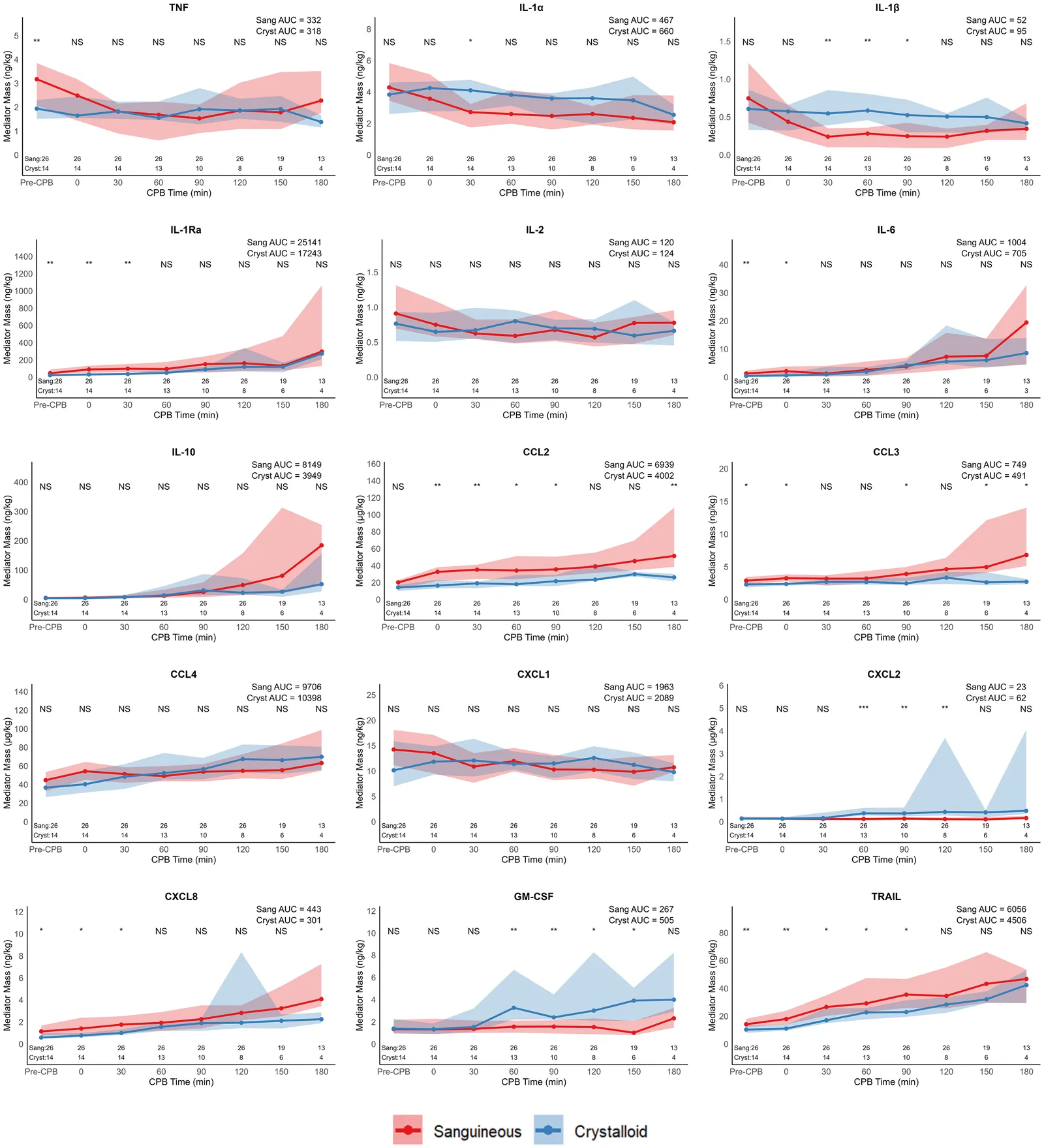
Cytokine and chemokine mediator mass are similar during CPB following sanguineous and crystalloid prime. Values are displayed as median and interquartile range ribbon. Statistical comparisons between groups are by the Wilcoxon rank sum test at each time point: NS indicates *P* > .05, * indicates *P* < .05, ** indicates *P* < .01, and ***indicates *P* < .001. Observations (N) through the time series are recorded. Abbreviations: AUC, area under curve; CCL, CC chemokine ligand; CPB, cardiopulmonary bypass; Cryst, crystalloid prime group; CXCL, CXC chemokine ligand; ET1, endothelin-1; GM-CSF, granulocyte-macrophage colony-stimulating factor; IL, interleukin; Sang, sanguineous prime group.

**Figure 3.**
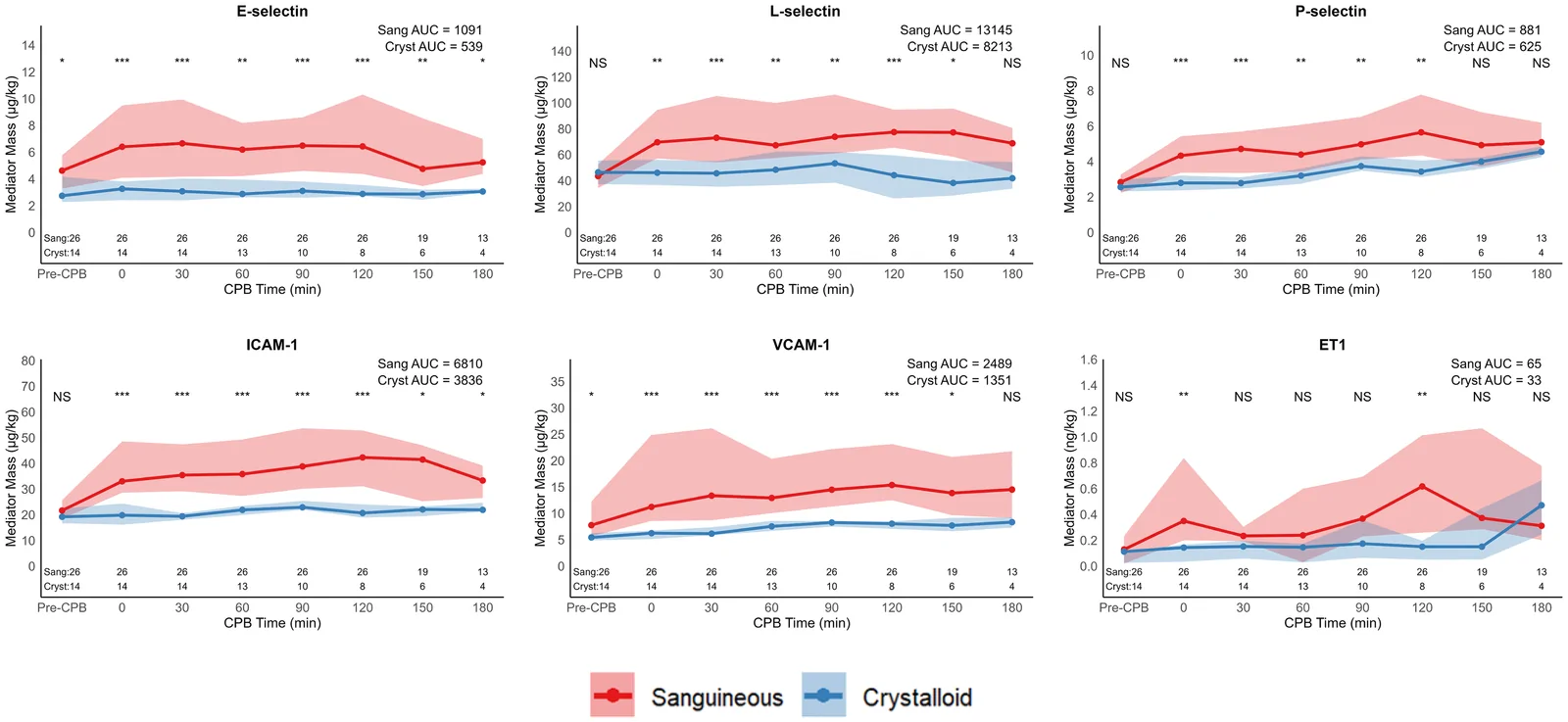
Adhesion molecule masses are elevated during CPB following sanguineous prime exposure. Values are displayed as median and interquartile range ribbon. Statistical comparisons between groups are by the Wilcoxon rank sum test at each time point: NS indicates *P* > .05, * indicates *P* < .05, ** indicates *P* < .01, and *** indicates *P* < .001. Observations (N) through the time series are recorded. Abbreviations: AUC, area under curve; CPB, cardiopulmonary bypass; Cryst, crystalloid prime group; ICAM-1, intracellular adhesion molecule 1; Sang, sanguineous prime group; VCAM-1, vascular cell adhesion molecule 1.

Patient-specific FC, between pre-CPB baseline and final reading on CPB, were calculated for each mediator and each group expressed as median fold change [95% CI] with complement mediators depicted in Figure 4 and cytokines, chemokines, and adhesion molecules in Figure 5. The *sanguineous prime* group had significantly larger median FC of C1q, C2, C3, C4, C4b, C5, C5a, CFB, CFH, CFI, L-selectin, and P-selectin (*P* < .05). C3a and C3b split products did not have a median fold change difference between groups. The *crystalloid prime* had significantly higher median FC of CXCL1 and CXCL2 (*P* < .05). TNF, IL-1α, IL-1β, and IL-2 had median fold decreases, and the *sanguineous prime* patients had a significantly larger median fold decrease relative to the crystalloid group (*P* < .05). Median FC of IL-1Ra, IL-6, IL-10, CCL2, CCL3, CCL4, CXCL8, GM-CSF, TRAIL, ET1, E-selectin, ICAM-1, and VCAM-1 were not different between groups (*P* > .05).

**Figure 4.**
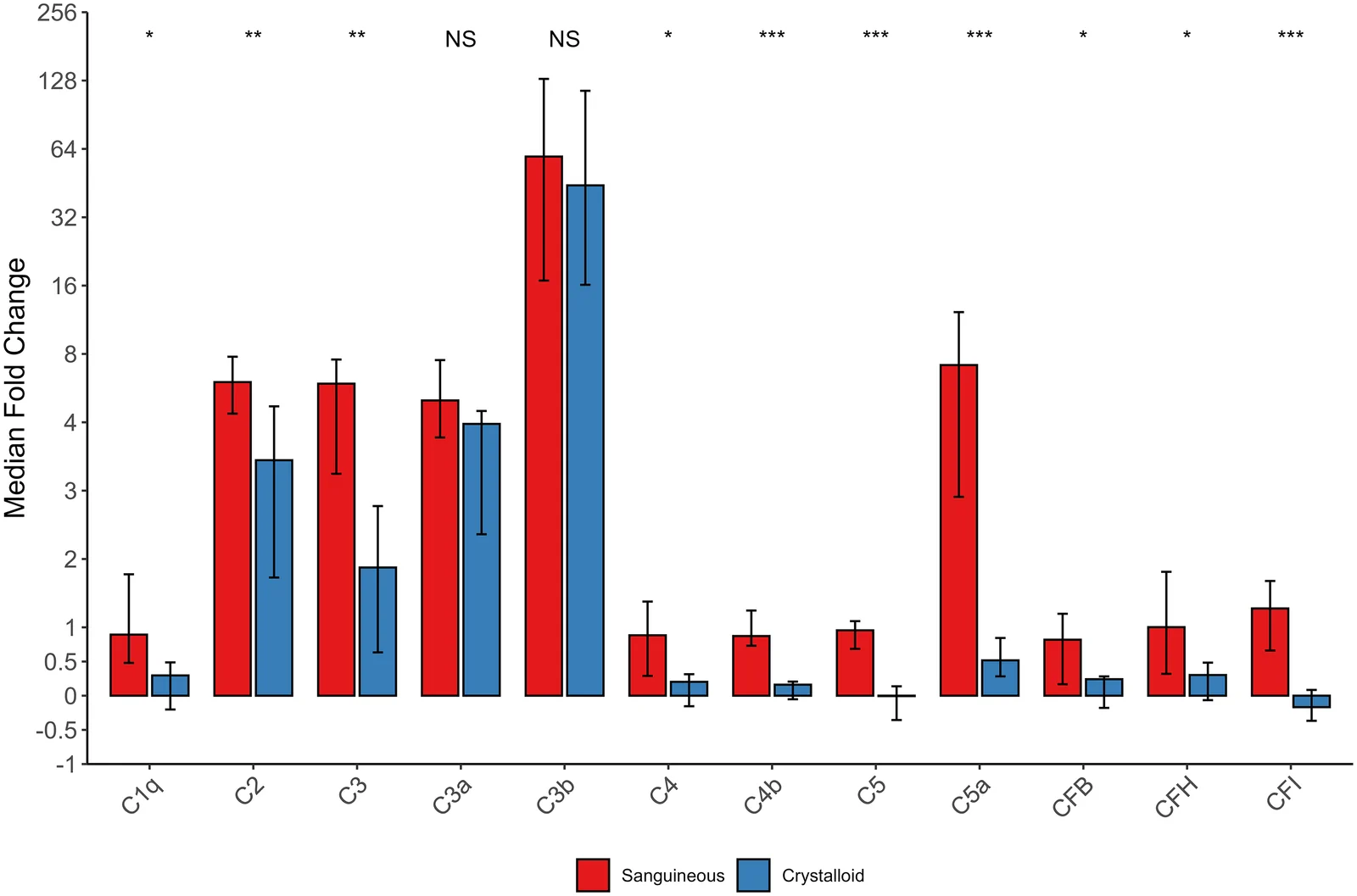
Complement mediator median fold change [95% CI] over CPB exposure. Statistical comparison is by the Wilcoxon rank sum test of fold changes between *sanguineous* (n = 26) and *crystalloid prime* (n = 14) patients. NS indicates *P* > .05, * indicates *P* < .05, ** indicates *P* < .01, and ***indicates *P* < .001. Abbreviations: C, complement; CF, complement factor; NS, not significant.

**Figure 5.**
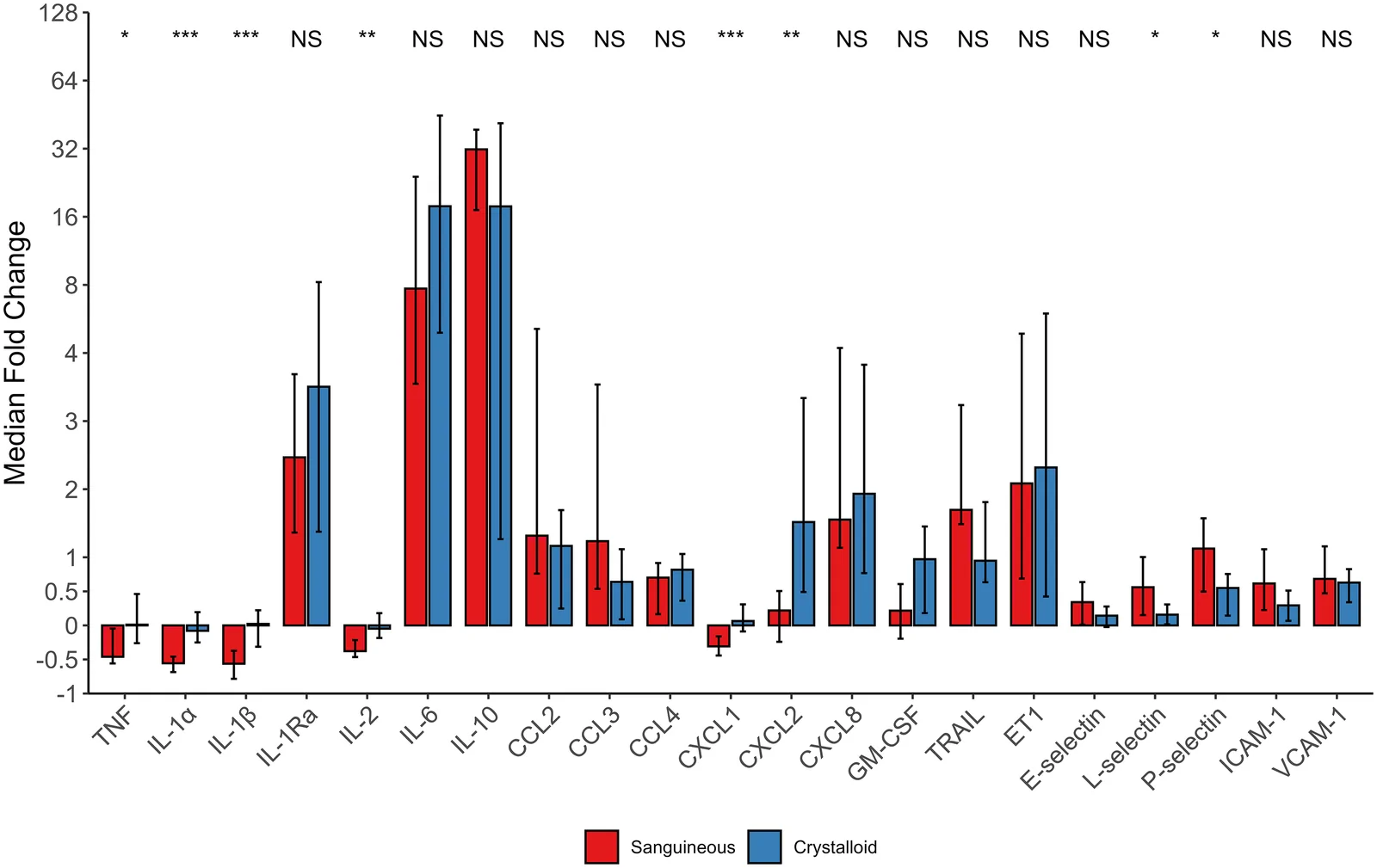
Cytokine, chemokine and adhesion molecule median fold change [95% CI] over CPB exposure. Statistical comparison is by the Wilcoxon rank sum test of fold changes between sanguineous (n = 26) and crystalloid patients (n = 14). NS indicates *P* > .05, * indicates *P* < .05, ** indicates *P* < .01, and ***indicates *P* < .001. Abbreviations: CCL, CC chemokine ligand; CXCL, CXC chemokine ligand; ET-1, endothelin-1; GM-CSF, granulocyte-macrophage stimulating factor; ICAM-1, intracellular adhesion molecule 1; IL, interleukin; NS, not significant. TNF, tumor necrosis factor; TRAIL, tumor necrosis factor-related apoptosis-inducing ligand; VCAM-1, vascular cell adhesion molecule 1.

A summary of mediator profiles by time series comparison and fold change is summarized in Table 3. There was congruence between time series and fold change assessment of complement mediators, excluding C3a and C3b split products, as well as L-selectin and P-selectin, with the *sanguineous prime* group showing a higher mediator burden. The *crystalloid prime* group showed a higher burden of CXCL2 by time series and fold change methods. There were some differential findings in cytokines and chemokines, with the *sanguineous prime* group showing a larger burden of CCL2, CCL3, and TRAIL by time series but not fold change assessment. The *crystalloid prime* group had a larger burden of TNF, IL-1α, IL-1β, IL-2, and CXCL1 by fold change, but this result was not observed in the time series.

**Table 3.** Summary of Comparisons.

Mediator	Time series	Fold change
**Complement**		
C1q (mg/kg)	Sanguineous > Crystalloid	Sanguineous > Crystalloid (*P* = .03)
C2 (mg/kg)	Sanguineous > Crystalloid	Sanguineous > Crystalloid (*P* = .007)
C3 (mg/kg)	Sanguineous > Crystalloid	Sanguineous > Crystalloid (*P* = .003)
C3a (ug/kg)	Sanguineous > Crystalloid	NS
C3b (mg/kg)	Sanguineous > Crystalloid	NS
C4 (mg/kg)	Sanguineous > Crystalloid	Sanguineous > Crystalloid (*P* = .02)
C4b (mg/kg)	Sanguineous > Crystalloid	Sanguineous > Crystalloid (*P* < .001)
C5 (mg/kg)	Sanguineous > Crystalloid	Sanguineous > Crystalloid (*P* < .001)
C5a (ng/kg)	Sanguineous > Crystalloid	Sanguineous > Crystalloid (*P* < .001)
CFB (mg/kg)	Sanguineous > Crystalloid	Sanguineous > Crystalloid (*P* = .04)
CFH (mg/kg)	Sanguineous > Crystalloid	Sanguineous > Crystalloid (*P* = .03)
CFI (mg/kg)	Sanguineous > Crystalloid	Sanguineous > Crystalloid (*P* < .001)
**Cytokines**		
TNF (ng/kg)	NS	Crystalloid > Sanguineous (*P* = .04)
IL-1α (ng/kg)	NS	Crystalloid > Sanguineous (*P* < .001)
IL-1β (ng/kg)	NS	Crystalloid > Sanguineous (*P* < .001)
IL-1Ra (ng/kg)	NS	NS
IL-2 (ng/kg)	NS	Crystalloid > Sanguineous (*P* = .009)
IL-6 (ng/kg)	NS	NS
IL-10 (ng/kg)	NS	NS
ET1 (ng/kg)	NS	NS
GM-CSF (ng/kg)	Crystalloid > Sanguineous	NS
TRAIL (ng/kg)	Sanguineous > Crystalloid	NS
**Chemokines**		
CCL2 (ug/kg)	Sanguineous > Crystalloid	NS
CCL3 (ng/kg)	Sanguineous > Crystalloid	NS
CCL4 (ug/kg)	NS	NS
CXCL1 (ng/kg)	NS	Crystalloid > Sanguineous (*P* < .001)
CXCL2 (ug/kg)	Crystalloid > Sanguineous	Crystalloid > Sanguineous (*P* = .001)
CXCL8 (ng/kg)	NS	NS
**Adhesion Molecules**		
E-selectin (ug/kg)	Sanguineous > Crystalloid	NS
L-selectin (ug/kg)	Sanguineous > Crystalloid	Sanguineous > Crystalloid (*P* = .04)
P-selectin (ug/kg)	Sanguineous > Crystalloid	Sanguineous > Crystalloid (*P* = .04)
ICAM-1 (ug/kg)	Sanguineous > Crystalloid	NS
VCAM-1 (ug/kg)	Sanguineous > Crystalloid	NS

Abbreviations: C, complement; CF, complement factor; CCL, CC chemokine ligand; CPB, cardiopulmonary bypass; CXCL, CXC chemokine ligand; ET1, endothelin-1; GM-CSF, granulocyte-macrophage colony-stimulating factor; ICAM-1, intracellular adhesion molecule 1; IL, interleukin; TNF, tumor necrosis factor; TRAIL, tumor necrosis factor-related apoptosis-inducing ligand; VCAM-1, vascular cell adhesion molecule 1.

## Discussion

Sanguineous preparation of CPB prime for pediatric cardiac surgery is associated with a significant complement burden and elevated quantities of soluble adhesion molecules that persist throughout CPB exposure. Cytokine and chemokine mediator masses were more similar between *sanguineous* and *crystalloid prime* groups, as the sanguineous prime did not contain relevant amounts of these inflammatory mediators. The unique complement dynamics following sanguineous prime exposure can be largely attributed to the supraphysiologic complement concentrations found in the FFP and preparation process.^6,13^ Upon CPB initiation, this complement-saturated sanguineous prime drives up the circulating mediator mass in neonates and infants, relative to those receiving a complement-free crystalloid prime. Sanguineous preparation of the prime could potentially be a critical influence on CPB-associated inflammation, most relevant to neonates and infants who experience the highest rates of morbidity following congenital cardiac surgery, although this requires further dedicated research to examine this concept as a modifiable clinical risk factor.^7,8,12,25^

Patients in both groups exhibit a robust alternative complement activation from their native innate immune system, although the reaction appears accelerated for those receiving a sanguineous prime. The tick-over mechanism activates C3 upon contact with the foreign non-endothelialized circuit, which initiates the alternative pathway cascade.^26,27^ The resulting split products, C3a and C3b, are thereby generated with multifocal proinflammatory actions, including endothelial cell activation and formation of the C3 and C5 convertase complexes, promoting further complement amplification.^9,26,27^ Hypothetically, there should be consistent production of these split products, which is observed in the time series of C3b but less so of C3a, which is flat over the CPB exposure in both groups. This could be potentially explained by continuous ultrafiltration used in all patients, which is known to extract C3a effectively and not C3b, or differential metabolism of the split products.^28,29^ It is noteworthy that C3a and C3b concentrations in each group converge at 180 min and 150 min of CPB, respectively. C5a mediator mass is significantly higher with sanguineous prime assessed by both time series and fold change. The *sanguineous prime* group exhibits a mediator mass spike at CPB initiation, which may correspond to the known C5a load associated with these types of prime preparations.^13^ C5a shares many similar anaphylatoxin functions as C3a, although it is more potent, and both complement products have been associated with morbidity and prolonged recovery following children's heart surgery.^11^

The adhesion molecules E-selectin, L-selectin, P-selectin, ICAM-1, and VCAM-1 were detected in the sanguineous CPB prime at concentrations similar to patient baselines. In contrast, they are absent in the clear crystalloid prime. Following CPB initiation, they share a similar profile to complement mediators and contrast with most cytokines and chemokines. Remarkably, the difference in adhesion molecule mediator mass between groups was largely sustained, and perhaps even grew throughout the CPB time following sanguineous prime exposure, with similar observed curve profiles throughout CPB as the complement mediators. These soluble adhesion molecules are known to reflect endothelial cell activation and even injury during CPB and sepsis.^30,31^ The associated observation of complement and adhesion molecules representing endothelial inflammation is difficult to ignore, as the postoperative inflammatory morbidities are systemic and involve multiple tissue beds.^8,9,32^ Although this study cannot assess differences in clinical outcomes between these 2 patient groups, future research might investigate the clinical implications of these immunologic findings.

Cytokine and chemokine profiles were relatively similar between *sanguineous* and *crystalloid prime,* with a few exceptions. The proinflammatory mediators TNF, IL-1α, and IL-1β showed minimal activity in both groups. IL-6 and CXCL8 were stimulated relatively equally, in accordance with prior research, and were not different between groups.^8,9^ The important anti-inflammatory mediator IL-10 was produced throughout CPB and did not differ between prime groups, indicating a robust pathway towards immune homeostasis.^33^ Therefore, it appears that the type of CPB prime has a more muted impact on cytokines and chemokines relative to the robust complement reaction. Specific mediators such as IL-1Ra, IL-6, IL-10, CCL2, CCL3, CCL4, CXCL2, and CXCL8 seem to rapidly accelerate towards the end of the time series in all patients. This observation might be related to myocardial ischemia after median cross-clamp removal times of 132 min and 92 min in the *sanguineous* and *crystalloid prime* groups, although further dedicated analysis is required.

Congenital cardiac specialists have historically assumed that neonates have an increased inflammatory response to CPB due to heightened postoperative inflammatory syndromes.^8,34^ However, other studies and reviews suggest that neonates have lower baseline complement levels than older patients and adults, have reduced chemotaxis, phagocytosis and decreased antigen presentation due to a relatively “immature” immune system.^35–37^ Therefore, a discrepancy exists between the observed postoperative clinical courses of neonates and their baseline immunologic milieu; further explanations are required. Because of the clear temporal relation between the sanguineous prime complement mediator concentrations and the immediate exaggerated rise of the same mediator concentrations in sanguinous prime patients, this exposure requires further investigation as a potentially modifiable risk factor for CPB-associated inflammation in neonates and small infants.

Naturally, the question then becomes how to optimize the CPB prime best, considering both inflammation and coagulation. Neonates are known to have multifocal coagulation deficiencies including hyporeactive platelets, reduced concentrations of vitamin-k dependent coagulation factors, and hypofibrinogenemia which can translate surgical bleeding.^38,39^ Sanguineous versus non-sanguineous CPB prime has been frequently studied in the context of the coagulation system and bleeding complications following pediatric cardiac surgery. Several small, randomized studies have investigated forms of FFP-free prime utilizing albumin. Consistently, hypofibrinogenemia was identified in the albumin primes and the albumin prime patients tended to receive more FFP transfusion postoperatively, although bleeding complications were not different.^1,4,40,41^ A single trial detected increased bleeding when FFP was administered after CPB versus in the CPB prime as 24 h chest drain output was significantly higher in the late FFP group (mean difference of 9 ml/kg [95% CI: 1-19] (*P* = .03)) as well as severe bleeding (defined as output over 30 ml/kg in the first 24 h) with 46% of late FFP patients versus 20% of early FFP patients (*P* = .02).^41^ Therefore, future innovation might consider CPB primes that reduce the inflammatory burden yet prevent hypofibrinogenemia and any associated bleeding.

## Limitations

Despite the comprehensiveness of this report in its description of inflammatory mediator changes throughout pediatric CPB, its limitations should be considered. The major constraint to this observational study is the inherent differences of demographic and operative characteristics between the 2 groups, as the sanguineous prime exposure of interest was applied only to patients less than 10 kg according to the institutional standard of care. To facilitate interpretations of intraoperative immunological results germane to this study's objective, and best mitigate this limitation at hand, several analytic approaches were utilized. First, inflammatory mediator concentrations were standardized on a per kilogram basis. Second, the measurements were restricted to the first 180 min of CPB prime and circuit exposure. Third, to account for the differences between CPB and myocardial ischemia time within the 180-min time frame, 2 complementary statistical approaches were used (time series and AUC). Fourth, a within-individual fold change approach was used as an adjunct to account for patient heterogeneity. Importantly, the time series, AUC, and fold change approaches were largely concordant. Other minor limitations also exist. Continuous ultrafiltration throughout CPB, and although this effect is constant and equal between both groups, the immunologic profiles might differ in the absence of this therapy. Finally, these results arise from a single institution and generalizability to other cardiac surgery and perfusion protocols is not certain.

Allowing for these limitations and mitigation strategies, there is substantial evidence that neonates and infants, because of the sanguineous prime exposure and relative mismatch between circulating blood volume and CPB circuit volume, experience exaggerated levels of activated complement relative to children receiving crystalloid prime. This burden is isolated only to the complement and leukocyte adhesion family of molecules, rather than cytokines and chemokines, which directly correlate to the sanguineous prime mediator measurements. Therefore, because sanguineous prime prepared with FFP contains a substantial complement load, and largely lacks cytokines and chemokines, the results here presented are biologically plausible.^6^ Furthermore, there is a temporal relationship between sanguineous prime exposure and complement biomarker increase observed immediately at the time of CPB initiation. Neonatal and infant patients in the *sanguineous prime* group immediately exhibited elevated complement concentrations, before the influence of intraoperative hypothermia, circulatory arrest, transfusion or extended CPB duration. Nevertheless, the results of this retrospective analysis require confirmation by prospective studies and direct comparison between similar patient groups.

## Conclusion

Patients who received sanguineous CPB prime were observed to carry a significant complement burden—particularly the activated mediators C3a, C3b, and C5a—and endothelial inflammation denoted by soluble adhesion molecule concentrations throughout CPB when compared with patients exposed to a clear crystalloid prime. In contrast, cytokine and chemokine profiles were broadly similar throughout CPB between patients exposed to sanguineous and crystalloid prime. Therefore, these allogeneic blood product preparations can be considered an immunogenic stimulus during pediatric CPB. Future innovation should focus on developing and evaluating inert alternatives for circuit prime and understanding the resulting impact on clinical outcomes after children's heart surgery.

## Supplemental Material

sj-docx-1-pch-10.1177_21501351261418297 - Supplemental material for The Potent Complement Stimulus of Sanguineous Versus Crystalloid Cardiopulmonary Bypass Prime during Pediatric Cardiac Surgery Supplemental material, sj-docx-1-pch-10.1177_21501351261418297 for The Potent Complement Stimulus of Sanguineous Versus Crystalloid Cardiopulmonary Bypass Prime during Pediatric Cardiac Surgery by Joel David Bierer, Roger Stanzel, Mark Henderson, John Sapp, Pantelis Andreou, Jean S. Marshall and David Horne in World Journal for Pediatric and Congenital Heart Surgery

## Abbreviations

AUC: area under the curve
C: complement
CF: complement factor
CI: confidence interval
CCL: C-C motif chemokine ligand
CXCL: C-X-C motif chemokine ligand
CPB: cardiopulmonary bypass
ET1: endothelin-1
FC: fold change
FFP: fresh frozen plasma
GM-CSF: granulocyte-macrophage colony-stimulating factor
ICAM-1: intercellular adhesion molecule 1
IL: interleukin
RBC: red blood cell
STAT: Society of Thoracic Surgeons-European Association for Cardio-Thoracic Surgery
TNF: tumor necrosis factor
TRAIL: tumor necrosis factor-related apoptosis-inducing ligand
TS: time series
VCAM-1: vascular cell adhesion molecule 1
