# Supplementary material for: The Potent Complement Stimulus of Sanguineous Versus Crystalloid Cardiopulmonary Bypass Prime during Pediatric Cardiac Surgery
Source: World J Pediatr Congenit Heart Surg. 2026 Mar 14;17(6):791–802. doi: 10.1177/21501351261418297 (PMC13624339; doi:10.1177/21501351261418297)
Supplement: Supplementary file 1 [file sj-docx-1-pch-10.1177_21501351261418297.docx]

**Supplement Table 1**

STROBE Statement—Checklist of items that should be included in reports of ***cohort studies.***

|  | | Item No | Recommendation | Page No |
| --- | --- | --- | --- | --- |
| **Title and abstract** | | 1 | (*a*) Indicate the study’s design with a commonly used term in the title or the abstract | NA |
|  |  |  | (*b*) Provide in the abstract an informative and balanced summary of what was done and what was found | 5 |
| Introduction | | | | |
| Background/rationale | | 2 | Explain the scientific background and rationale for the investigation being reported | 7 |
| Objectives | | 3 | State specific objectives, including any prespecified hypotheses | 7 |
| Methods | | | | |
| Study design | | 4 | Present key elements of study design early in the paper | 8 |
| Setting | | 5 | Describe the setting, locations, and relevant dates, including periods of recruitment, exposure, follow-up, and data collection | 8 |
| Participants | | 6 | (*a*) Give the eligibility criteria, and the sources and methods of selection of participants. Describe methods of follow-up | 9 |
|  |  |  | (*b*) For matched studies, give matching criteria and number of exposed and unexposed | NA |
| Variables | | 7 | Clearly define all outcomes, exposures, predictors, potential confounders, and effect modifiers. Give diagnostic criteria, if applicable | 8 |
| Data sources/ measurement | | 8* | For each variable of interest, give sources of data and details of methods of assessment (measurement). Describe comparability of assessment methods if there is more than one group | 8 |
| Bias | | 9 | Describe any efforts to address potential sources of bias | 9 |
| Study size | | 10 | Explain how the study size was arrived at | 8 |
| Quantitative variables | | 11 | Explain how quantitative variables were handled in the analyses. If applicable, describe which groupings were chosen and why | 10 |
| Statistical methods | | 12 | (*a*) Describe all statistical methods, including those used to control for confounding | 10 |
|  |  |  | (*b*) Describe any methods used to examine subgroups and interactions | NA |
|  |  |  | (*c*) Explain how missing data were addressed | 7 |
|  |  |  | (*d*) If applicable, explain how loss to follow-up was addressed | 7 |
|  |  |  | (*e*) Describe any sensitivity analyses | 7 |
| Results | | | |  |
| Participants | | 13* | (a) Report numbers of individuals at each stage of study—eg numbers potentially eligible, examined for eligibility, confirmed eligible, included in the study, completing follow-up, and analysed | 12 |
|  |  |  | (b) Give reasons for non-participation at each stage | 12 |
|  |  |  | (c) Consider use of a flow diagram | NA |
| Descriptive data | | 14* | (a) Give characteristics of study participants (eg demographic, clinical, social) and information on exposures and potential confounders | 12 |
|  |  |  | (b) Indicate number of participants with missing data for each variable of interest | 12 |
|  |  |  | (c) Summarise follow-up time (eg, average and total amount) | 15 |
| Outcome data | | 15* | Report numbers of outcome events or summary measures over time | 13-15 |
| Main results | 16 | (*a*) Give unadjusted estimates and, if applicable, confounder-adjusted estimates and their precision (eg, 95% confidence interval). Make clear which confounders were adjusted for and why they were included | | 13-15 |
|  |  | (*b*) Report category boundaries when continuous variables were categorized | | NA |
|  |  | (*c*) If relevant, consider translating estimates of relative risk into absolute risk for a meaningful time period | | NA |
| Other analyses | 17 | Report other analyses done—eg analyses of subgroups and interactions, and sensitivity analyses | | NA |
| Discussion | | | | |
| Key results | 18 | Summarise key results with reference to study objectives | | 16 |
| Limitations | 19 | Discuss limitations of the study, taking into account sources of potential bias or imprecision. Discuss both direction and magnitude of any potential bias | | 19 |
| Interpretation | 20 | Give a cautious overall interpretation of results considering objectives, limitations, multiplicity of analyses, results from similar studies, and other relevant evidence | | 19 |
| Generalisability | 21 | Discuss the generalisability (external validity) of the study results | | 19 |
| Other information | | | | |
| Funding | 22 | Give the source of funding and the role of the funders for the present study and, if applicable, for the original study on which the present article is based | | 2 |
